# REPLY TO THE AUTHORS: Re: Long term outcomes of onestage augmentation anterior urethroplasty: a systematic review and meta-analysis

**DOI:** 10.1590/S1677-5538.IBJU.2022.0474.1

**Published:** 2022-11-20

**Authors:** Cooper R. Benson, Gen Li, Steven B. Brandes

**Affiliations:** 1 Columbia University Medical Center Department of Urology New York NY USA Department of Urology, Columbia University Medical Center, New York, NY, USA; 2 Columbia University Medical Center Department of Biostatistics New York NY USA Department of Biostatistics, Columbia University Medical Center, New York, NY, USA

*To the editor*,

We appreciate the editorial comments on our manuscript (1, 2). Unfortunately, the reconstructive urology urethroplasty literature is almost all retrospective and cohort sizes are relatively small. Moreover, only a few papers report interim or long-term follow up success. It is because of the very limitations and weaknesses of the urethroplasty literature that we needed to perform a systematic review and meta-analysis. We closely followed the PRISMA method here and understand the critique of not utilizing the Newcastle-Ottawa Scale to assess the studies. The main take home of our analysis is that the “85% success rate” that is often quoted preoperatively to patients appears to be a clear over -estimation of the success of augmentation urethroplasty. Despite the inherent limitations of the literature, our manuscript clearly shows that augmentation urethroplasty has a slow and progressive recurrence rate overtime. The longer the follow up the more recurrences are identified. Augmentation urethroplasty demonstrates good success at intermediate follow-up, but with longer follow up it appears that it is not the panacea that it is commonly thought.

The authors.
